# Validity evidence for the Brazilian version of the Postpartum Specific Anxiety Scale

**DOI:** 10.1590/0034-7167-2024-0268

**Published:** 2025-07-11

**Authors:** Kydja Milene Souza Torres de Araújo, Jaqueline Wendland, Rosilene Santos Baptista

**Affiliations:** I Universidade de Pernambuco. Recife, Pernambuco, Brazil; II Universidade de Paris. Île-de-France, France; III Universidade Estadual da Paraíba. Campina Grande, Paraíba, Brazil

**Keywords:** Postpartum Period, Anxiety, Mental Health, Health Promotion, Validation Study., Periodo Posparto, Ansiedad, Salud Mental, Promoción de la Salud, Estudio de Validación.

## Abstract

**Objectives:**

to analyze the validity evidence of the Brazilian version of the Postpartum Specific Anxiety Scale.

**Methods:**

psychometric study carried out with 262 women in the first six months after giving birth. The analysis included: item-total correlation, exploratory factor analysis, confirmatory factor analysis, Cronbach’s alpha and test-retest. Parallel analysis was used to adjust dimensionality. Evidence of the validity of relationships with other variables was verified through the correlation between the PSAS, the Edinburgh Postpartum Depression scale and the Trait-State Anxiety Inventory.

**Results:**

the removal of eight items improved the quality of fit of the baseline model without affecting reliability. The results indicated stability and evidence of the validity of relationships with other variables showed positive correlations between the PSAS and the other scales used for this purpose.

**Conclusions:**

the Postpartum Specific Anxiety Scale has satisfactory psychometric parameters for the Brazilian context.

## RESUMEN

**Objetivos:**

analizar las evidencias de validez de la versión brasileña de la Escala de Detección de Ansiedad Posparto (PSAS).

**Métodos:**

es un estudio psicométrico realizado entre 262 mujeres durante los primeros seis meses del posparto. El análisis incluyó: correlación ítem-total, análisis factorial exploratorio, análisis factorial confirmatorio, alfa de Cronbach y test-retest. Se utilizó el análisis paralelo para ajustar la dimensión y se comprobó la validez de las relaciones con otras variables mediante la correlación entre la PSAS, la Escala de Depresión Posparto de Edimburgo y el Inventario de Ansiedad Rasgo-Estado.

**Resultados:**

la eliminación de ocho ítems mejoró la calidad de ajuste del modelo referencial sin afectar la fiabilidad. Los resultados indicaron estabilidad y corroboraron la validez de las relaciones con otras variables, demostrando así, correlaciones positivas entre la PSAS y las demás escalas utilizadas para este fin.

**Conclusiones:**

la Escala de Detección de Ansiedad Posparto exhibe parámetros psicométricos satisfactorios para el contexto brasileño.

## INTRODUCTION

Some women experience the postpartum period with great enthusiasm and others find it devastating, a time when they are predisposed to emotional fragility^(1,2)^. The most prevalent mental disorder at this stage of life is baby blues or puerperal sadness^(3)^. However, postpartum anxiety has become a subject of interest for researchers in the perinatal field. It is important to emphasize that perinatal mental disorders cannot be related only to postpartum depression, since women at this stage of life can present various other mental health problems, such as phobias, post-traumatic stress disorder, postpartum psychosis and anxiety^(4)^. For this reason, it is suggested that the term “postpartum depression” be replaced by “perinatal mood and anxiety disorders”^(5)^.

Global studies indicate that anxiety in the first six months after childbirth has a prevalence of 6.1% to 40%, which is considered higher than postpartum depression. Some factors may have influenced this variation: the instrument used, the severity of the symptoms, the country and the period in which the assessment was carried out^(6-8)^. However, research into this condition in Brazil seems to be less extensive, since no specific and validated instrument has been identified in the national literature for this purpose. Considering this data, it is essential that the signs and symptoms of postpartum anxiety are correctly identified and measured^(9)^, as it has been associated with negative effects on infant temperament, sleep, mental development and health, harmful changes in the bond between mother and baby, reduced breastfeeding, decreased maternal self-efficacy, adverse results in infant feeding, exacerbation of depression in women with a previous diagnosis and increased risk of suicide^(7,10-14)^.

In 2014, the National Institute for Health and Care Excellence in England called for attention to the under-detection of this condition^(15)^, as it is understood that the mother’s mental health directly affects the baby’s well-being and can also influence marital and family relationships, thus making its investigation, diagnosis and treatment necessary^(16)^. In Brazil, Bill PL 702/2015 was drafted, which provides for the psychological assessment of pregnant women during prenatal care and puerperal women between 48 hours and 15 days after childbirth.

In this sense, considering the need for a valid and reliable instrument that can contribute to the assessment of anxiety in the postpartum period, the Postpartum Specific Anxiety Scale (PSAS) was identified, developed and validated in 2016 in England by Fallon et al.^(10)^ and cross-culturally adapted for Brazil by Araújo et al.^(17)^. Considering that the PSAS is a specific instrument for measuring anxiety in the first six months after childbirth, it is understood that its use in Brazil could provide subsidies for clinical practice as well as for the field of research, since it is an unprecedented instrument.

## OBJECTIVES

To analyze the validity evidence of the Brazilian version of the Postpartum Specific Anxiety Scale (PSAS).

## METHODS

### Ethical aspects

Study approved by the Research Ethics Committee. Authorization was obtained from the authors of the scale to carry out the study and the Informed Consent Form was signed by all the participants (written signature).

### Study design, period and location

Psychometric study carried out between September 2021 and February 2022 that analyzed the internal structure and relationship with other variables of the Postpartum Specific Anxiety Scale for Brazil, after obtaining authorization from Dr. Vicky Fallon, author of the original version. The study was carried out in two health centers in the municipality of Recife (PE), Brazil. The population was composed of all the mothers who came to vaccinate their babies up to six months old or to have the Newborn blood spot test. The eligibility criteria were: women aged 18 or over who were experiencing the first six months after giving birth.

The sample size was determined according to Pasquali’s recommendation^(18)^, which states that five to ten respondents are needed per item, and that this number should not be less than 200. In this sense, considering that the original version of the PSAS includes 51 items, the sample consisted of 262 women according to the inclusion criteria. These women also took part in the analysis of evidence of the validity of relationships with other variables. Of these 262 women, fifty took part in the test-retest stage, which was the ideal number for this analysis^(19)^.

### Study protocol

Data collection was carried out by the author of the study and two nursing students who had previously been introduced to the objectives and instruments used: a sociodemographic questionnaire (age, marital status, schooling, family income and family arrangement), the Edinburgh Postpartum Depression Scale (EPDS) and the State-Trait Anxiety Inventory (STAI). Given that the health centers where the study was carried out offer vaccination and Newborn blood spot test services on a spontaneous basis, women arriving with their babies were randomly invited to take part in the research. The interview took place individually and lasted an average of 40 minutes. All participants were presented with the objectives of the study and signed the informed consent form.

In a second step, the PSAS was reapplied in the retest stage. In order to reach 50 women for the retest, all participants were asked at the first meeting to provide their telephone number so that the retest could be carried out on the fourteenth day after the first contact with the interviewer. Thus, considering an interval of 10 to 14 days to be appropriate^(20)^, the retest was carried out by telephone 14 days after the first application of the PSAS, thus respecting the interval used in the original study and by other authors in other cultures^(10,21,22)^.

### Data analysis

Initially, a database was built in EXCEL 2016 and double-checked by another researcher. Once the spreadsheet had been checked, the data was transferred to a definitive database using the Statistical Package for the Social Sciences (SPSS), version 26.0.0.0. For psychometric analysis, the EFA and CFA were carried out using IBM SPSS Amos 26.0.0 software, and Parallel Analysis using Factor software, version 12.01.02.

Measures of central tendency (simple frequency, mean, maximum and minimum) and measures of dispersion (standard deviation) were used to analyze and describe the sociodemographic data. The education and age variables were categorized according to the classification of the Brazilian Institute of Geography and Statistics (IBGE)^(23)^ and income was based on the minimum wage in force in 2021 (the year the study began). Before starting the Factor Analysis (FA), the correlation matrix was checked using Spearman’s coefficient, due to the non-normality of the sample identified by the Shapiro-Wilk test. This made it possible to verify the association between the 51 items of the instrument studied. After checking the correlation matrix, it was decided to follow Damásio’s recommendation^(24)^: to apply the KMO measure to check that the PA model is adequately adjusted to the data and Bartlett’s Test of Sphericity to check for correlations between the variables.

After checking the basic assumptions for using factor analysis, the domain extraction stage began. At this stage, the number of domains was determined and those to be extracted were first defined. This was initially done using the “eigenvalue” criterion (>1) and the Scree test. We then opted to use Parallel Analysis, with the aid of Factor software, version 12.01.02, based on minimum rank factor analysis^(25)^, to confirm the real number of domains that the instrument has. Parallel analysis generated random and uncorrelated data (set of matrices) and compared them with the eigenvalues of the exploratory factor analysis. This simulated data was factored and the average of the eigenvalues from this simulation was calculated. In this way, the domain extraction stage was completed and then the domains were rotated using the Varimax orthogonal rotation technique using the principal component method.

To confirm the viability of the model created by the exploratory factor analysis, Confirmatory Factor Analysis (CFA) was carried out using the following measures: Chi-square Mean/Degree of Freedom (CMIN/DF)^(26)^, Quality of Fit Index (GFI)^(27)^, Root Mean Square Residual (RMSR)^(28)^, Root Mean Square Error of Approximation (RMSEA)^(29)^, Comparative Fit Index (CFI)^(30)^, Adjusted Fit Quality Index (AGFI)^(29)^. After defining the model using the CFA, the cut-off point was defined using the percentile curve. Thus, the P25 and P50 percentiles were considered for calculation, as they are more central, and so the sample was divided into three groups (< P25, P25 to P75 and >P75) which correspond to three quartiles, with Q1 = P7532,33. Thus, considering that P25 means that 25% of the individuals in the sample are on the left and 75% on the right, and so on, the values corresponding to Q1 were classified as “mild”, for Q2 “moderate” and for Q3 “high”, according to Duran’s study^(21)^.

In addition to the Exploratory and Confirmatory Factor Analyses, the reliability of the version of the PSAS adapted for written and spoken Portuguese in Brazil was analyzed through internal consistency, using Cronbach’s alpha, and temporal stability, through test-retest using Spearman’s correlation coefficient. As for evidence of the validity of relationships with other variables, in this study this was done by correlating the focal instrument (the PSAS) with two others that assess a similar construct: the Edinburgh Postpartum Depression Scale - EPDS^(31)^ and the State-Trait Anxiety Inventory - STAI^(32)^. Spearman’s correlation coefficient was used for this stage.

## RESULTS

A total of 262 women took part, aged between 18 and 46 years (mean 29.3 years). There was a predominance of women who declared themselves to be brown (46.2%), Catholic (39.7%), married or with a partner (82.1%), with a level of education equal to or greater than 11 years (53.1%) and a total family income of one to two minimum wages (41.2%). The mean income of the sample was R$1,474.58 from salaries, but the amounts reported ranged from R$0.00 to R$22,000.00.

Analysis of the correlation matrix between all the items showed the strongest correlations between items 24 and 25 (p=0.628) and that most of the correlations were positive and above 0.3. The result of KMO = 0.816 indicated that the sample size was adequate to carry out the factor analysis. Bartlett’s Test of Sphericity provided information on the presence of correlations between the original variables, and in this study it showed a value of (ꭓ2=4009.087; p<0.05), i.e. the p-value test was approximately zero, so Bartlett’s test was statistically significant, with a significance level of 5%, thus indicating that the correlation matrix was different from the identity. Therefore, the database adequacy criteria were met, concluding the database adequacy verification stage.

According to the eigenvalue criterion, the maximum number of domains was 16, but the Scree test showed that the number suggested was 5. Therefore, considering the technique that most restricted the number of domains in this study (Scree test), solutions with up to five domains were considered in order to find the model that was most understandable and similar to the original. In this sense, the possibilities of acceptable arrangements were tested, considering the slope diagram (Scree test) and the eigenvalue criterion (eigenvalue), i.e. (1 to 5 domains) were tested, using Varimax orthogonal rotation, using the principal component method. With five domains, the eigenvalue of the last domain was only 1.78.

When considering the psychometric and theoretical indicators, it was observed that the most appropriate number was four domains, since most of the items are organized in these domains and it coincides with the number of domains in the original scale. Finally, four domains were retained, which explain 31.692% of the variance in the model. The parallel analysis showed that in the first four domains, the empirical eigenvalues were greater than the random eigenvalues, so the model with four domains proved to be adequate, and these are called: G1 - Competence and attachment anxieties; G2 - Anxiety about the baby’s safety and well-being; G3 - Care practice anxieties; G4 - Psychosocial adjustment to motherhood.

Varimax orthogonal rotation using the principal component method, with four domains, revealed that five items (8, 11, 22, 32 and 44) had factor loadings below 0.3. These items were therefore excluded and the model readjusted. The loadings were extracted again and the results after Varimax rotation are shown in Table 1. As in the original PSAS construction and validation study, the cross-loaded items were chosen according to the criterion of theoretical congruence. In this sense, item 31, loaded in groups 3 and 4, was retained in group 3; item 38, loaded in groups 1, 2 and 4, was retained in group 4; and item 39, loaded in groups 1, 3 and 4, was also retained in group 4.

**Table 1:** Rotated component matrix after excluding items with factor loadings < 0.30, Recife, Pernambuco, Brazil, 2024

Item	Component				Item	Component			
	1	2	3	4		1	2	3	4
1	**.475**	.270	-.068	.103	27	.102	.006	.171	**.322**
2	**.407**	-.067	.280	-.026	28	-.026	.082	**.562**	.297
3	.154	.113	.053	**.582**	29	**.558**	.309	.090	.151
4	.025	**.491**	-.067	.206	30	**.547**	-.092	.131	.149
5	**.481**	.152	-.075	.111	31	-.069	.226	**.375**	**.384**
6	**.482**	.310	.030	.119	33	-.074	.139	.258	**.574**
7	.244	**.350**	.201	.031	34	.271	.005	.203	**.421**
9	.035	**.588**	-.074	.393	35	.272	.056	-.039	**.333**
10	**.574**	.038	.033	-.051	36	.000	**.426**	.241	-.274
12	**.550**	.089	.109	.084	37	**.388**	.090	.046	.156
13	.101	.041	**.581**	.157	38	**.306**	**.341**	-.010	**.366**
14	.247	.153	**.448**	.167	39	**.315**	.296	**.307**	**.316**
15	.070	.079	.237	**.541**	40	.218	.180	**.588**	.061
16	.140	-.022	.104	**.655**	41	.161	.009	**.569**	.069
17	.031	**.471**	.121	.110	42	**.322**	.128	.116	.233
18	.143	**.594**	.122	.353	43	**.587**	.039	-.024	.016
19	**.472**	-.049	.235	.138	45	.237	**.385**	.213	.107
20	.196	**.528**	.129	-.016	46	**.568**	.161	.136	.020
21	**.420**	.197	.099	-.211	47	.066	.148	**.635**	.099
23	.216	.128	**.492**	.246	48	**.502**	.151	.076	-.032
24	.211	**.643**	.333	-.017	49	-.050	.129	**.550**	.104
25	.328	**.616**	.127	-.100	50	-.068	.116	.214	**.455**
26	**.387**	.019	.170	.106	51	**.432**	.233	.049	.141

It can be seen, then, that all the items were well distributed between the domains, thus maintaining the pattern previously identified: G1 - Competence and attachment anxieties (items 1, 2, 5, 6, 10, 12, 19, 21, 26, 29, 30, 37, 42, 43, 46, 48 and 51), which explained 18.604% of the variance; G2 - Anxiety about baby’s safety and well-being (items 4, 7, 9, 17, 18, 20, 24, 25, 36, and 45) which explained 6.392% of the variance; G3 - Care practice anxieties (items 13, 14, 23, 28, 31, 40, 41, 47 and 49) which explained 4.386% of the variance and G4 - Psychosocial adjustment to motherhood (items 3, 15, 16, 27, 33, 34, 35, 38, 39 and 50) which explained 4.089% of the variance, with the total variance explained being 33.471%.

**Figure 1:**
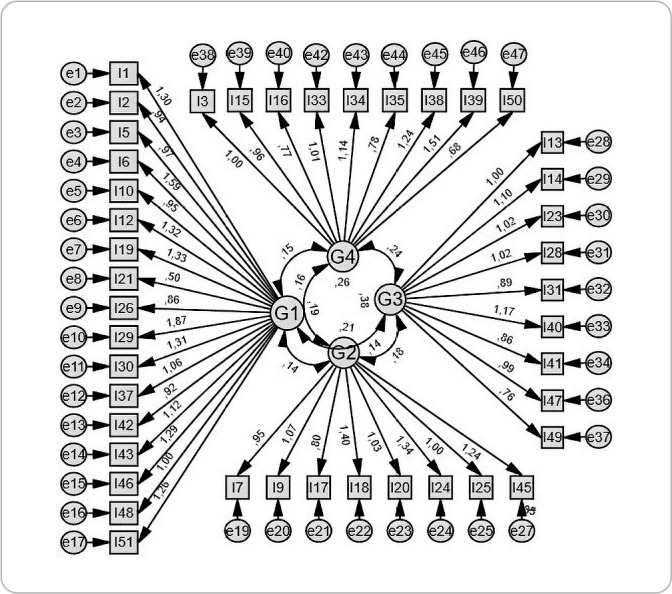
Graphical representation of the Confirmatory Factor Analysis of the PSAS after elimination of items with standardized regression weight < 0.3, Recife, Pernambuco, Brazil, 2024

The result of the first confirmatory analysis for all items revealed that the standardized regression weight of three items was below 0.3: ITEM 4 (G2), ITEM 27 (G4) and ITEM 36 (G2). For this reason, they were removed from the scale, leaving 43 items (Figure 1), which were again submitted to a new confirmatory factor analysis.

Thus, in the new model found, the items had a standardized regression weight above 0.30. The new model also showed a good RMSEA fit (p<0.05): 0.034. Chi-square 1079.365 (p=.000), degree of freedom = 825(=1079.365; p<0.05; /df=1.308). SRMR= 0.0578; CFI= 0.90; NFI= .0.69; GFI=.85; AGFI= 0.83. Thus, after the new confirmatory factor analysis, it was found that the Brazilian version of the PSAS comprises 43 items distributed in four domains.

The results indicated an overall Cronbach’s alpha of over 0.70, which was also achieved for each domain (Table 2).

**Table 2:** Cronbach’s alpha overall and by domain of the Postpartum Specific Anxiety Scale Brazilian version, Recife, Pernambuco, Brazil, 2024

Domain	CRONBACH'S ALPHA	Nº of items
G1 - Competence and attachment anxieties	0.82	17
G2 - Anxiety about baby's safety and well-being	0.75	8
G3 - Care practice anxieties	0.78	9
G4 - Psychosocial adjustment to motherhood	0.73	9
**Overall**	**0.90**	**43**

In the test-retest analysis of temporal stability, the results showed a total value of the correlations maintained of r = 0.928 with p ≤ 0.01. Table 3 shows the correlation values for each domain, all of which were statistically significant.

**Table 3:** Spearman’s correlation coefficient values of the test-retest by domain of the Postpartum Specific Anxiety Scale Brazilian version, Recife, Pernambuco, Brazil, 2024

	Correlation
G1 - Competence and attachment anxieties	0.848^*^
G2 - Anxiety about baby's safety and well-being	0.664^*^
G3 - Care practice anxieties	0.870^*^
G4 - Psychosocial adjustment to motherhood	0.845^*^
**Overall**	0.928^*^

* *The correlation is significant at the 0.01 level.*

In the analysis of evidence of the validity of relationships with other variables using Spearman’s correlation, the results showed a positive and statistically significant correlation between the Brazilian version of the PSAS and the State-Trait Anxiety Inventory (STAI), divided into two subscales that assess anxiety as a “state” (STAI-E) or “trait” (STAI-T), and the Edinburgh Postpartum Depression Scale (EPDS), thus indicating that the version of the PSAS adapted for Brazil presents convergent evidence (Table 4).

**Table 4:** Spearman’s correlation coefficient values in the evidence of validity of relationships with other variables in the Brazilian version of the Postpartum Specific Anxiety Scale, Recife, Pernambuco, Brazil, 2024

	EPDS	STAI - E	STAI - T
**PSAS Total**	.419^^**^^	.515^^**^^	.534^^**^^

PSAS: Postpartum Specific Anxiety Scale; STAI: State-Trait Anxiety Inventory; STAI-E: divided into two subscales that assess anxiety as a “state”; STAI-T: divided into two subscales that assess anxiety as a “trait”; EPDS: Edinburgh Postpartum Depression Scale.^**^The correlation is significant at the 0.01 level.

## DISCUSSION

The results of this study show that the structure with four correlated domains was confirmed, which shows that they refer to the same construct and that the adaptation construct for the Brazilian context presented similar components to the original version developed in England. A similar result was found in the structure of the PSAS after validation for France^(22)^ and Iran^(33)^, also showing that the Brazilian construct may be similar to the construct adapted for these countries, differing only from the construct adapted for Turkey, where the PSAS, after validation, was formatted in a one-dimensional structure^(21)^. The CFA determined the adapted model with 43 items. It should be noted that the original version consists of 51 items. However, validation with the removal of items also took place during the PSAS validation process for France, where a version with 44 items was generated^(22)^; for Turkey, a version with 47 items was reproduced^(21)^ and for Iran, a version with 49 items^(33)^.

A reduced version with 16 items (Postpartum Specific Anxiety Scale Research Short-Form - PSAS-RSF) was developed and validated^(9)^ and a rapid response version with 12 items (Postpartum Specific Anxiety Scale Research Short-Form for use in global crises - PSAS-RSF-C) was also developed and validated for use during the COVID-19 pandemic in England, since the National Institute for Health and Care Excellence in that country published guidelines stating that during the pandemic, measurement instruments should contain fewer than 12 items in order to optimize accessibility^(34)^. In the original study to develop and validate the PSAS^(10)^, all 51 items achieved a factor load of at least 0.30. However, in the version adapted for Brazil, eight items (4, 8, 11, 22, 27, 32, 36 and 44) did not reach this value, and these items were excluded during the exploratory and confirmatory factor analyses.

The low correlation of item 4 “I’ve been worried about how to deal with my baby when others aren’t around to support me” may be related to the ease of communication via mobile phone or even access to information in the palm of your hand using the internet, which means that when in doubt, puerperal women can ask for guidance or help via messaging apps, phone calls, video calls and even health professionals via the virtual environment. This ease of communication and access to information can have a positive influence, so that the puerperal woman doesn’t feel insecure when she is alone with her baby.

In addition, studies have shown that WhatsApp is one of the most effective tools for remote health care^(35-37)^, as it allows groups to be formed by people who share the same goal, as well as individual care via text message, voice message or even video call. There is also the use of social networks such as Instagram, Facebook and blogs. These possibilities have great educational potential, since they contribute to interpersonal relationships, thus favoring the exchange of experiences and the construction of collective learning^(38)^.

The low correlation of items 8 “I have felt scared when my baby is not with me”, 11 “I worry that my baby will feel better under someone else’s care”, 32 “I have felt that when I receive help it is not beneficial”, 36 “I have felt that my baby would be better cared for by someone else” and 44 “I have found it difficult to sleep even when I have the chance” may be related to the presence of a support network, which, in the study by Nóbrega et al.^(36)^, the most influential members were the spouse (father) and mother (grandmother).

Considering postpartum women, this support can be provided by family, friends, neighbors and health professionals^(39,40)^. In this way, considering the feelings that exist among the members of the support network, it can be said that the woman who receives this support will probably feel secure about caring for her baby and the kind of help she receives, as well as feeling confident about her relationship with her baby, even if the baby is also being cared for by someone else, and will be able to enjoy moments of rest and relaxation, however short these periods may be.

Regarding items 22 “I have thought of ways to avoid exposing my baby to germs” and 27 “I have not participated in daily activities with my baby because I fear he may hurt himself”, the low correlation may be related to the fact that data collection took place during the COVID-19 pandemic. Thus, in view of the pandemic scenario, all Brazilian states and municipalities, as well as all countries around the world, have adopted and started to recommend protective measures for all individuals of all ages and in any clinical health condition, among which we can mention: social isolation (only leaving the house for what is necessary), social distancing, mandatory use of a mask in all environments, hand hygiene^(41)^. In this sense, considering the health guidelines and recommendations, it is likely that participation in daily activities was influenced by the health restrictions and not by the fear of the baby getting hurt. What’s more, these protective measures have become a mandatory part of people’s daily lives on all continents, thus becoming habitual and not just for babies.

The overall alpha value was 0.90, a result very close to those found by other researchers who have worked with the same scale in other countries: 0.96, 0.93, 0.91, 0.93^(10,22,21,33)^. In this sense, it is understood that the translated and adapted PSAS is reliable and internally consistent for use in Brazilian culture, as well as in other cultures such as France, Iran, Turkey and the UK. The results obtained from the test-retest analysis showed that there was consistency in the repetitions of the answers in the PSAS adapted for Brazil. In this study, a correlation coefficient of 0.928 was obtained, which was higher than the original version (0.8810^(10)^) and the French version (0.8439^(22)^), but similar to the Iranian and Turkish versions^(21,33)^. It can therefore be said that the PSAS adapted for Brazil is not only consistent and reliable, but also stable over time^(20,42)^.

The values from the convergent validity analysis showed positive and statistically significant correlations between the adapted version of the PSAS for Brazil and the questionnaires used. Among the analyses, the strongest correlation was found with the STAI-T (trait) form (0.515 - significant correlation at the 0.01 level). This assesses general disposition to anxiety, while the STAI-E (state) assesses situational anxiety^(22)^. Similar results were also found in the study by Fallon et al.^(10)^ and Duran^(21)^ and, in addition to indicating good convergent validity, may be associated with the fact that anxiety in the postpartum period could be related to an exacerbation of psychological vulnerabilities, such as vulnerability to anxiety itself, given the multiple psychological adjustments that motherhood brings with it^(43)^. The positive correlation with the EPDS confirms that anxiety and depression can coexist in the postpartum period^(10,44)^.

### Study limitations

Considering the sample of this study, it is recognized that a limitation may be related to the ratio between the number of observations and variables, which was 5:1. Although this ratio meets the minimum recommendation of 5 minimum observations per variable^(18)^, it is at the lower limit, as the literature suggests a sample of 500 or more cases, whenever possible, to guarantee reliable decisions with a minimum of 200 respondents^(45,46)^. This aspect can generate another limitation related to the generalization of the results since the ability to generalize the results to a larger population can be limited if the sample is not large enough to capture the variability in the population. The authors recognize as another possible limitation the fact that the retest was carried out by telephone, since telephone interviews are subject to interruptions and background noise which can affect the quality of communication, as well as technical problems such as poor signal quality, dropped calls or inadequate equipment which can compromise the quality of the telephone interview.

### Contributions to the fields of nursing, health or public policy

Investigating anxiety in the postpartum period is a necessary intervention. In this sense, the provision of a specific tool for this purpose could contribute to improvements in the process of identifying this problem and bring advances in research, thus contributing to better planning of mental health care in the postpartum period.

## CONCLUSIONS

The Brazilian version of the Postpartum Specific Anxiety Scale (PSAS-BR) showed evidence of validity of the internal structure. The PSAS-BR can therefore be considered a suitable and useful tool for use in clinical practice to investigate anxiety in the first six months after childbirth. In this sense, it is considered that this instrument could be relevant for use in the context of mental health in the puerperium, as it could benefit the practice of health professionals in terms of health promotion, especially nurses who deal daily and continuously with this public.
