# Supplementary material for: Validity evidence for the Brazilian version of the Postpartum Specific Anxiety Scale
Source: Rev Bras Enferm. 2025 Jul 11;78(3):e20240268. doi: 10.1590/0034-7167-2024-0268 (PMC12252928; doi:10.1590/0034-7167-2024-0268)
Supplement: Translation

# Translation: Evidências de validade da versão brasileira da *Postpartum Specific Anxiety Scale*

**DOI:** 10.1590/0034-7167-2024-0268pt

**Authors:** Kydja Milene Souza Torres de Araújo, Jaqueline Wendland, Rosilene Santos Baptista

**Affiliations:** I Universidade de Pernambuco. Recife, Pernambuco, Brasil; II Universidade de Paris. Île-de-France, França; III Universidade Estadual da Paraíba. Campina Grande, Paraíba, Brasil

**Keywords:** Período Pós-Parto, Ansiedade, Saúde Mental, Promoção da Saúde, Estudo de Validação.

## Abstract

**Objetivos:**

analisar as evidências de validade da versão brasileira da *Postpartum Specific Anxiety Scale*.

**Métodos:**

estudo psicométrico realizado com 262 mulheres nos primeiros seis meses após o parto. A análise incluiu: correlação item-total, análise fatorial exploratória, análise fatorial confirmatória, Alfa de Cronbach e teste-reteste. Para ajuste de dimensionalidade foi utilizada a análise paralela. A evidência de validade de relações com outras variáveis foi verificada através da correlação entre a PSAS, a escala de Depressão pós-parto de Edimburgo e o Inventário de Ansiedade traço-estado.

**Resultados:**

a eliminação de oito itens melhorou a qualidade de ajuste do modelo basal sem afetar a fidedignidade. Os resultados indicaram estabilidade e a evidência de validade de relações com outras variáveis mostrou correlações positivas entre a PSAS e as demais escalas utilizadas para este fim.

**Conclusões:**

a *Postpartum Specific Anxiety Scale* apresenta parâmetros psicométricos satisfatórios para o contexto brasileiro.

## INTRODUÇÃO

Algumas mulheres experienciam o período pós-parto com grande entusiasmo enquanto outras podem considerá-lo devastador, sendo esse um momento de predisposição à fragilidade emocional^(1,2)^. O transtorno mental mais prevalente nessa etapa da vida é o Baby-blues ou tristeza puerperal^(3)^. Porém, a ansiedade pós-parto tem se tornado assunto de interesse para pesquisadores da área perinatal. É fundamental destacar que os transtornos mentais perinatais não podem ser relacionados apenas à depressão pós-parto, pois mulheres nessa mesma etapa da vida podem apresentar diversos outros problemas no âmbito da saúde mental, como fobias, transtorno do estresse pós-traumático, psicose pós-parto e ansiedade^(4)^. Por esse motivo, sugere-se a substituição do termo “depressão pós-parto” para “distúrbios perinatais de humor e ansiedade”^(5)^.

Estudos globais indicam que a ansiedade nos primeiros seis meses após o parto tem uma prevalência de 6,1% a 40%, sendo considerada superior à depressão pós-parto. Alguns fatores podem ter influenciado na variação: instrumento que foi utilizado, a severidade dos sintomas, país e período onde foi realizada a avaliação^(6-8)^. Contudo, a pesquisa sobre tal condição no Brasil parece ser menos extensa, uma vez que não foi identificado, na literatura nacional, um instrumento específico e validado para este fim. Considerando esse dado, é essencial que os sinais e sintomas de ansiedade no pós-parto sejam corretamente identificados e mensurados^(9)^, pois ela tem sido associada a efeitos negativos no temperamento infantil, no sono, no desenvolvimento mental e na saúde, a alterações prejudiciais no vínculo entre mãe e bebê, redução do aleitamento materno, diminuição da autoeficácia materna, resultados adversos na alimentação infantil, exacerbação da depressão naquelas mulheres com diagnóstico prévio e aumento do risco de suicídio^(7,10-14)^.

Em 2014, o Instituto Nacional para Saúde e Cuidados de Excelência na Inglaterra solicitou a atenção para a subdetecção desse agravo^(15)^, pois compreende-se que a saúde mental da mãe afeta diretamente o bem-estar do bebê e pode também influenciar no relacionamento conjugal e familiar, fazendo-se, assim, necessários sua investigação, diagnóstico e tratamento^(16)^. No Brasil, foi elaborado o Projeto de Lei PL 702/2015, que dispõe sobre a avaliação psicológica de gestantes durante o pré-natal e puérperas entre 48h e 15 dias após o parto.

Nesse sentido, considerando-se a necessidade de um instrumento válido e confiável que possa contribuir com a avaliação da ansiedade no pós-parto, foi identificada a *Postpartum Specific Anxiety Scale* (PSAS), desenvolvida e validada em 2016 na Inglaterra por Fallon et al.,^(10)^ e adaptada transculturalmente para o Brasil por Araújo et al.^(17)^. Ao considerar que a PSAS é um instrumento específico para mensurar a ansiedade nos primeiros seis meses após o parto, compreende-se que seu uso no Brasil poderá fornecer subsídios para a prática clínica bem como para o campo da pesquisa, uma vez que se trata de um instrumento inédito.

## OBJETIVOS

Analisar as evidências de validade da versão brasileira da Postpartum Specific Anxiety Scale (PSAS).

## MÉTODOS

### Aspectos éticos

Estudo aprovado pelo Comitê de Ética em Pesquisa. A autorização dos autores da escala para realização do estudo foi obtida e o Termo de Consentimento Livre e Esclarecido foi assinado por todas as participantes (assinatura escrita).

### Desenho, período e local do estudo

Trata-se de um estudo psicométrico, realizado entre setembro de 2021 a fevereiro de 2022, que analisou a estrutura interna e a relação com outras variáveis da *Postpartum Specific Anxiety Scale* para o Brasil, após obtenção de autorização com a Drª Vicky Fallon, autora da versão original. O estudo foi desenvolvido em duas unidades de saúde no município de Recife (PE), Brasil. A população foi composta por todas as mães que chegassem vacinar seus bebês de até seis meses de vida ou para realizar o teste do pezinho. Os critérios de elegibilidade foram: mulheres com idade igual ou superior a 18 anos e que estavam vivenciando os primeiros seis meses após o parto.

Para determinação do tamanho da amostra, considerou-se a recomendação de Pasquali^(18)^, o qual orienta que são necessários de cinco a dez sujeitos respondentes por item, não devendo este quantitativo ser inferior a 200 indivíduos. Nesse sentido, considerando que a versão original da PSAS contempla 51 itens, a amostra foi composta por 262 mulheres, de acordo com os critérios de inclusão. Estas também participaram da análise da evidência de validade de relações com outras variáveis. Dessas 262 mulheres, cinquenta participaram da etapa do teste-reteste, sendo esse o quantitativo ideal para essa análise^(19)^.

### Protocolo do estudo

A coleta dos dados foi realizada pela autora do estudo e duas acadêmicas de enfermagem previamente apresentadas aos objetivos e instrumentos utilizados: questionário sociodemográfico (idade, estado civil, escolaridade, renda familiar e arranjo familiar), Escala de Depressão Pós-parto de Edimburgo (EPDS) e Inventário de Ansiedade Traço-Estado (IDATE). Considerando que as unidades de saúde onde o estudo foi desenvolvido contemplam os serviços de vacinação e teste do pezinho com demanda espontânea, as mulheres que chegavam com seus bebês eram convidadas aleatoriamente a participar da pesquisa. A entrevista ocorreu individualmente e teve duração média de 40 minutos. A todas as participantes foram apresentados os objetivos do estudo e todas assinaram o TCLE.

Em um segundo momento, a PSAS foi reaplicada na etapa do reteste. Para que fosse alcançado o quantitativo de 50 mulheres para o reteste, solicitou-se, no primeiro encontro, a todas as participantes que fornecessem o contato telefônico, no décimo quarto dia após o primeiro contato com a entrevistadora, que o reteste fosse realizado. Assim, considerando-se como adequado o intervalo de 10 a 14 dias^(20)^, o reteste foi realizado por contato telefônico 14 dias após a primeira aplicação da PSAS, respeitando-se, assim, o intervalo utilizado no estudo original e por demais autores em outras culturas^(10,21,22)^.

### Análise dos dados

Inicialmente, construiu-se um banco de dados no EXCEL 2016 que passou por dupla conferência por outro (a) pesquisador (a). Com a planilha conferida, os dados foram transportados para um banco de dados definitivos, por meio do *Statistical Package for the Social Sciences* (SPSS), versão 26.0.0.0. Para análise psicométrica realizou-se a AFE e AFC com uso do *software* IBM SPSS Amos 26.0.0, a Análise Paralela com auxílio do *software* Factor, versão 12.01.02.

Para análise e descrição dos dados sociodemográficos, utilizou-se as medidas de tendência central (frequência simples, média, máximo e mínimo) e medidas de dispersão (desvio padrão). As variáveis escolaridade e idade foram categorizadas de acordo com a classificação do Instituto Brasileiro de Geografia e Estatística (IBGE)^(23)^ e a renda foi baseada no salário-mínimo vigente em 2021 (ano de início da pesquisa). Antes de se iniciar a Análise Fatorial (AF), realizou-se a verificação da matriz de correlações, por meio do coeficiente de *Spearman*, devido à não normalidade da amostra identificada pelo Teste de Shapiro-Wilk. Com isso, foi possível verificar a associação entre os 51 itens do instrumento estudado. Após a verificação da matriz de correlações, optou-se por seguir a recomendação de Damásio^(24)^: aplicar a medida KMO para verificar se o modelo da AF está ajustado adequadamente aos dados, e o Teste de Esfericidade de Bartlett para averiguar a presença de correlações entre as variáveis.

Após verificação das suposições-base para uso da análise fatorial, iniciou-se a etapa de extração de domínios. Nessa etapa, realizou-se a determinação do número de domínios, sendo primeiramente definidos aqueles que seriam extraídos. Essa definição foi realizada, inicialmente, pelo critério do “autovalor (*eigenvalue*)” (>1) e do diagrama de inclinação (*Scree test*). Em seguida, optou-se pela utilização da Análise Paralela, com auxílio do *software* Factor, versão 12.01.02, baseada no minimum rank factor analysis^(25)^, para confirmar o número real de domínios que o instrumento apresenta. A análise paralela gerou dados aleatórios (conjunto de matrizes) e não correlacionados e os comparou com os autovalores da análise fatorial exploratória. Esses dados simulados foram fatorados e a média dos autovalores desta simulação foi calculada. Desta forma, finalizou-se a etapa de extração dos domínios e, em seguida, realizou-se a rotação destes, utilizando-se da técnica de rotação ortogonal Varimax pelo método do componente principal.

Para confirmar a viabilidade do modelo originado na análise fatorial exploratória, realizou-se a Análise Fatorial Confirmatória (AFC) através das medidas: Chi-square Mean/Degree of Freedom (CMIN/DF)^(26)^, Índice de Qualidade de Ajuste (GFI)^(27)^, Raiz Quadrada do Resíduo Quadrático Médio (SRMR)^(28)^, Raiz do Erro Quadrático Médio de Aproximação (RMSEA)^(29)^, Índice de Ajuste Comparativo (CFI)^(30)^, Índice de Qualidade de Ajuste Ajustado (AGFI)^(29)^. Após definição do modelo a partir da AFC, realizou-se a definição do ponto de corte através da curva de percentis. Dessa forma, considerou-se para cálculo os percentis P25 e P50, por serem mais centrais e, assim, a amostra foi dividida em três grupos (< P25, P25 até P75 e >P75) que correspondem a três quartis, sendo Q1 = P7532,33. Assim, considerando que P25 significa que 25% dos indivíduos que compõem a amostra estão à esquerda e 75% à direita e, assim, sucessivamente, os valores correspondentes a Q1 foram classificados como “leve”, para Q2 “moderado” e para Q3 “elevado”, conforme o estudo de Duran^(21)^.

Além das Análises Fatorial Exploratória e Confirmatória, a fidedignidade da versão adaptada para o português escrito e falado no Brasil da PSAS foi analisada mediante a consistência interna, por meio do Alfa de Cronbach e da estabilidade temporal, por intermédio do teste-reteste utilizando-se o coeficiente de correlação de *Spearman*. Sobre as evidências de validade de relações com outras variáveis, neste estudo, esta foi realizada pela correlação do instrumento focal (a PSAS) com outros dois que avaliam um construto similar: Escala de Depressão Pós-parto de Edimburgo - EPDS^(31)^ e o Inventário de Ansiedade Traço-Estado - IDATE^(32)^. Para esta etapa, utilizou-se coeficiente de correlação de *Spearman*.

## RESULTADOS

Participaram 262 mulheres com idade que variou entre 18 e 46 anos (média de 29,3 anos). Houve predomínio das que se declararam pardas (46,2%), católicas (39,7%), casadas ou com companheiro (82,1%), nível de escolaridade com tempo de estudo igual ou superior a 11 anos (53,1%) e renda familiar total de um a dois salários-mínimos (41,2%). A renda média da amostra foi de R$ 1.474,58 proveniente de salário, contudo, os valores informados variaram de R$ 0,00 a R$ 22.000,00.

A análise da matriz de correlação entre todos os itens demonstrou correlações mais fortes entre os itens 24 e 25 (p=0,628) e que a maioria das correlações foram positivas e acima de 0,3. O resultado de KMO = 0,816 indicou que o tamanho amostral foi adequado para execução da análise fatorial. O Teste de Esfericidade de Bartlett forneceu informações sobre a presença de correlações entre as variáveis originais, tendo apresentado neste estudo valor de (=4009,087; p<0,05), ou seja, o teste do p-valor foi, aproximadamente, zero, logo, o teste de Bartlett foi estatisticamente significante, com nível de significância de 5%, indicando, assim, que a matriz de correlação foi diferente da identidade. Portanto, os critérios de adequação da base de dados foram atendidos, concluindo-se, assim, a etapa de verificação da adequação da base de dados.

Pelo critério de autovalor, observou-se o quantitativo máximo de 16 domínios, porém, pelo Diagrama de declividade (*Scree test*), constatou-se que o número sugerido foi de 5. Dessa forma, levando em conta a técnica que mais restringiu (*Scree test*) o número de domínios, neste estudo, considerou-se soluções com até cinco domínios para saber o modelo mais compreensível e semelhante ao original. Nesse sentido, testaram-se as possibilidades de arranjos aceitáveis, considerando o diagrama de declividade (*Scree test*) e critério do autovalor (*eigenvalue*), ou seja, foram testados (1 a 5 domínios), utilizando rotação ortogonal Varimax, pelo método componente principal. Com cinco domínios, o *eigenvalue* do último domínio foi de apenas 1,78.

Ao considerar os indicadores psicométricos e teóricos, observou-se que o número mais adequado foi de quatro domínios, uma vez que a maioria dos itens se encontra organizada dessa forma e que coincide com o número de domínios da escala original. Finalmente, quatro domínios foram retidos, os quais explicam 31,692% da variância do modelo. A análise paralela evidenciou que nos primeiros quatro domínios, os autovalores empíricos foram maiores do que o autovalores aleatórios, portanto, o modelo com quatro domínios mostrou-se adequado, sendo estes denominados: G1 - Competência e ansiedades de apego; G2 - Ansiedade de segurança e bem-estar do bebê; G3 - Ansiedades da prática do cuidado; G4 - Ajuste psicossocial à maternidade.

A rotação ortogonal Varimax pelo método componente principal, com quatro domínios, revelou que cinco itens (8, 11, 22, 32 e 44) apresentaram cargas fatoriais abaixo de 0.3. Portanto, esses itens foram excluídos e o modelo reajustado. As cargas foram extraídas novamente e os resultados após a rotação Varimax estão apresentados na Tabela 1. Assim como no estudo original de construção e validação da PSAS, a escolha dos itens de carga cruzada foi realizada de acordo com o critério de congruência teórica. Nesse sentido, o item 31, carregado nos grupos 3 e 4, ficou retido no grupo 3; o item 38, carregado nos grupos 1, 2 e 4, foi retido no grupo 4; e o item 39, carregado nos grupos 1, 3 e 4, foi retido também no grupo 4.

**Tabela 1:** Matriz de componente rotativa após exclusão dos itens com carga fatorial < 0,30, Recife, Pernambuco, Brasil, 2024

Item	Componentes				Item	Componentes			
	1	2	3	4		1	2	3	4
1	**,475**	,270	-,068	,103	27	,102	,006	,171	**,322**
2	**,407**	-,067	,280	-,026	28	-,026	,082	**,562**	,297
3	,154	,113	,053	**,582**	29	**,558**	,309	,090	,151
4	,025	**,491**	-,067	,206	30	**,547**	-,092	,131	,149
5	**,481**	,152	-,075	,111	31	-,069	,226	**,375**	**,384**
6	**,482**	,310	,030	,119	33	-,074	,139	,258	**,574**
7	,244	**,350**	,201	,031	34	,271	,005	,203	**,421**
9	,035	**,588**	-,074	,393	35	,272	,056	-,039	**,333**
10	**,574**	,038	,033	-,051	36	,000	**,426**	,241	-,274
12	**,550**	,089	,109	,084	37	**,388**	,090	,046	,156
13	,101	,041	**,581**	,157	38	**,306**	**,341**	-,010	**,366**
14	,247	,153	**,448**	,167	39	**,315**	,296	**,307**	**,316**
15	,070	,079	,237	**,541**	40	,218	,180	**,588**	,061
16	,140	-,022	,104	**,655**	41	,161	,009	**,569**	,069
17	,031	**,471**	,121	,110	42	**,322**	,128	,116	,233
18	,143	**,594**	,122	,353	43	**,587**	,039	-,024	,016
19	**,472**	-,049	,235	,138	45	,237	**,385**	,213	,107
20	,196	**,528**	,129	-,016	46	**,568**	,161	,136	,020
21	**,420**	,197	,099	-,211	47	,066	,148	**,635**	,099
23	,216	,128	**,492**	,246	48	**,502**	,151	,076	-,032
24	,211	**,643**	,333	-,017	49	-,050	,129	**,550**	,104
25	,328	**,616**	,127	-,100	50	-,068	,116	,214	**,455**
26	**,387**	,019	,170	,106	51	**,432**	,233	,049	,141

Percebe-se, então, que todos os itens se distribuíram bem entre os domínios, mantendo, assim, o padrão anteriormente identificado: G1 - Competência e ansiedades de apego (itens 1, 2, 5, 6, 10, 12, 19, 21, 26, 29, 30, 37, 42, 43, 46, 48 e 51), o qual explicou 18,604% da variância; G2 - Ansiedade de segurança e bem-estar do bebê (itens 4, 7, 9, 17, 18, 20, 24, 25, 36, e 45) que explicou 6,392% da variância; G3 - Ansiedades da prática do cuidado (itens 13, 14, 23,28, 31, 40, 41, 47 e 49 ) que explicou 4,386% da variância e o G4 - Ajuste psicossocial à maternidade (itens 3, 15, 16, 27, 33, 34, 35, 38, 39 e 50) que explicou 4,089% da variância, sendo o total da variância explicada de 33,471%.

O resultado da primeira análise confirmatória para todos os itens revelou que o *standardised regression weight* de três itens ficou abaixo de 0.3: ITEM 4 (G2), ITEM 27 (G4) e ITEM 36 (G2). Por essa razão, estes foram removidos da escala, permanecendo, assim, 43 itens (Figura 1), os quais foram novamente submetidos à nova análise fatorial confirmatória.

**Figura 1:** Representação gráfica da Análise Fatorial Confirmatória da PSAS após eliminação dos itens com *standardised regression weight* < 0.3, Recife, Pernambuco, Brasil, 2024

Assim, no novo modelo encontrado, os itens apresentaram um o *standardised regression weight* acima de 0.30. O novo modelo também apresentou bom ajuste RMSEA (p<0,05): 0,034. Chi-square 1079,365 (p=.000), degree of freedom = 825(=1079,365; p<0,05; 1,308). SRMR= 0.0578; CFI= 0.90; NFI= .0,69; GFI=.85; AGFI= 0,83. Assim, após a nova análise fatorial confirmatória, constatou-se que a versão brasileira da PSAS comporta 43 itens distribuídos em quatro domínios.

Os resultados indicaram um alfa de Cronbach geral superior a 0,70, sendo este o resultado também alcançado por cada domínio (Tabela 2).

**Tabela 2:** Alfa de Cronbach geral e por domínio da *Postpartum Specific Anxiety Scale* versão brasileira, Recife, Pernambuco, Brasil, 2024

Domínios	ALFA DE CRONBACH	Nº de itens
G1 - Competência e ansiedades de apego	0,82	17
G2 - Ansiedade de segurança e bem-estar do bebê	0,75	8
G3 - Ansiedades da prática do cuidado	0,78	9
G4 - Ajuste psicossocial à maternidade	0,73	9
**Geral**	**0,90**	**43**

Na análise da estabilidade temporal através do teste-reteste, os resultados mostraram um valor total das correlações mantidas de r = 0.928 com p ≤ 0,01. Na Tabela 3, encontram-se os valores de correlação para cada domínio, sendo todos estatisticamente significantes.

**Tabela 3:** Valores do coeficiente de correlação de *Spearman* do teste-reteste por domínio da *Postpartum Specific Anxiety Scale* versão brasileira, Recife, Pernambuco, Brasil, 2024

	Correlação
G1 - Competência e ansiedades de apego	0,848^*^
G2 - Ansiedade de segurança e bem-estar do bebê.	0,664^*^
G3 - Ansiedades da prática do cuidado.	0,870^*^
G4 - Ajuste psicossocial à maternidade.	0,845^*^
**Total**	0,928^*^

* *A correlação é significativa no nível 0,01.*

Na análise das evidências de validade de relações com outras variáveis através da correlação de *Spearman*, os resultados mostraram correlação positiva e estatisticamente significante entre a versão brasileira da PSAS e o Inventário de Ansiedade Traço-Estado (IDATE), dividido em duas subescalas que avaliam a ansiedade enquanto “estado” (IDATE-E) ou “traço” (IDATE-T), e a Escala de Depressão Pós-Parto de Edimburgo (EPDS), indicando, assim, que a versão adaptada da PSAS para o Brasil apresenta evidência convergente (Tabela 4).

**Tabela 4:** Valores do coeficiente de correlação de *Spearman* nas evidências de validade de relações com outras variáveis da *Postpartum Specific Anxiety Scale* versão brasileira, Recife, Pernambuco, Brasil, 2024

	EPDS	IDATE - E	IDATE - T
**PSAS Total**	,419^^**^^	,515^^**^^	,534^^**^^

PSAS: Postpartum Specific Anxiety Scale; IDATE: Inventário de Ansiedade Traço-Estado; IDATE-E: dividido em duas subescalas que avaliam a ansiedade enquanto “estado”; IDATE-T: dividido em duas subescalas que avaliam a ansiedade enquanto “traço”; EPDS: Escala de Depressão Pós-Parto de Edimburgo.^**^A correlação é significativa no nível 0,01.

## DISCUSSÃO

Os resultados deste estudo mostram que a estrutura com quatro domínios correlacionados entre si foi confirmada, o que evidencia que se referem ao mesmo constructo e que o construto da adaptação para o contexto brasileiro apresentou componentes similares à versão original desenvolvida na Inglaterra. Resultado semelhante foi encontrado na estrutura da PSAS após validação para a França^(22)^ e Irã^(33)^, evidenciando, também, que o constructo brasileiro pode apresentar semelhança ao constructo adaptado para esses países, havendo diferença apenas do constructo adaptado para a Turquia, em que a PSAS, após a validação, foi formatada em estrutura unidimensional^(21)^. A AFC determinou o modelo adaptado com 43 itens. Ressalta-se que a versão original é composta por 51 itens. No entanto, a validação com remoção de itens também ocorreu durante o processo de validação da PSAS para a França, em que foi gerada a versão com 44 itens^(22)^; para a Turquia, reproduziu-se a versão com 47 itens^(21)^ e para o Irã, a versão com 49 itens^(33)^.

Uma versão reduzida com 16 itens (*Postpartum Specific Anxiety Scale Research Short-Form* - PSAS-RSF) foi desenvolvida e validada^(9)^ e uma versão para resposta rápida com 12 itens (*Postpartum Specific Anxiety Scale Research Short-Form* for use in global crises - PSAS-RSF-C) foi também desenvolvida e validada para uso durante a pandemia da COVID-19 na Inglaterra, uma vez que o Instituto Nacional para Saúde e Cuidados de Excelência desse país publicou nas diretrizes que durante a pandemia, os instrumentos de medida deveriam conter menos de 12 itens, para que fosse otimizada a acessibilidade^(34)^. No estudo original de desenvolvimento e validação da PSAS^(10)^, todos os 51 itens alcançaram carga fatorial de, no mínimo, 0,30. No entanto, na versão adaptada para o Brasil, oito itens (4, 8, 11, 22, 27, 32, 36 e 44) não alcançaram esse valor, sendo estes os itens excluídos durante as análises fatoriais exploratória e confirmatória.

A baixa correlação do item 4 “Tenho me preocupado em como lidar com meu bebê quando outros não estiverem por perto para me apoiar” pode estar relacionada à facilidade de comunicação via telefone móvel ou até mesmo o acesso às informações na palma da mão com uso da internet, com isso, é possível que em um momento de dúvida, a mulher no puerpério possa pedir orientações ou ajuda por meio de aplicativos de mensagens, ligações, videochamadas e até mesmo realizar atendimento com profissional de saúde pelo ambiente virtual. Essa facilidade de comunicação e acesso à informação pode influenciar positivamente, para que a puérpera não se sinta insegura quando estiver sozinha com o bebê.

Além disso, estudos têm mostrado que o WhatsApp tem uma das ferramentas eficazes para o atendimento remoto à saúde^(35-37)^, pois permite a formação de grupos por pessoas que compartilham do mesmo objetivo, bem como possibilita o atendimento individual por mensagem de texto, de voz ou até videochamada. Além disso, também pode-se citar o uso das redes sociais, como Instagram, Facebook, além de blogs. Essas possibilidades apresentam grande potencial educativo, uma vez que contribuem para o relacionamento interpessoal, favorecendo, assim, a troca de experiências e a construção da aprendizagem coletiva^(38)^.

A baixa correlação dos itens 8 “Tenho me sentido assustada quando meu bebê não está comigo”, 11 “Me preocupo que meu bebê se sinta melhor sob os cuidados de outra pessoa”, 32 “Tenho sentido que quando recebo alguma ajuda ela não é benéfica”, 36 “Tenho sentido que meu bebê seria melhor cuidado por outra pessoa” e 44 “Tenho tido dificuldade para dormir mesmo quando tenho chance” pode estar relacionada ao fato da presença de uma rede de apoio, a qual, no estudo de Nóbrega et al.^(36)^, teve como integrantes mais influentes o cônjuge (pai) e a mãe (avó).

Considerando a mulher no pós-parto, esse apoio pode ser fornecido por familiares, amigos, vizinhos e profissionais de saúde^(39,40)^. Sendo assim, ao cogitar os sentimentos existentes entre os integrantes da rede de apoio, pode-se afirmar que a mulher que recebe esse suporte, provavelmente se sentirá segura quanto aos cuidados do bebê e ao tipo de ajuda que recebe, assim como se sentirá confiante em relação ao relacionamento com o bebê, ainda que o mesmo receba também cuidados de outra pessoa e poderá usufruir de momentos de descanso e relaxamento, por menores que sejam esses períodos.

Em relação aos itens 22 “Tenho pensado em maneiras de evitar a exposição do meu bebê a germes” e 27 “Não tenho participado de atividades cotidianas com meu bebê porquê temo que ele possa se machucar”, a baixa correlação pode estar relacionada ao fato de que a coleta dos dados ocorreu durante a pandemia da COVID-19. Assim, tendo em vista o cenário pandêmico, todos os estados e municípios brasileiros, bem como todos os países ao redor do mundo adotaram e passaram a recomendar medidas de proteção para todos os indivíduos de todas as idades e em qualquer condição clínica de saúde, dentre as quais, podem-se citar: isolamento social (somente sair de casa para o necessário), distanciamento social, uso obrigatório de máscara em todos os ambientes, higienização das mãos^(41)^. Nesse sentido, considerando as orientações e recomendações sanitárias, é provável que a participação em atividades cotidianas tenha sido influenciada pelas restrições sanitárias e não pelo medo do bebê se machucar. Além disso, essas medidas de proteção passaram a fazer obrigatoriamente parte do dia a dia das pessoas em todos os continentes, tornando-se, assim, algo habitual e não exclusivamente para bebês.

O valor geral de alfa foi de 0.90, sendo um resultado muito próximo aos encontrados por outros pesquisadores que trabalharam com a mesma escala em outros países: 0.96, 0.93, 0.91, 0.93^(10,22,21,33)^. Nesse sentido, compreende-se que a PSAS traduzida e adaptada apresenta-se confiável e consistente internamente para uso na cultura brasileira, bem como para outras culturas, como a francesa, iraniana, turca e britânica. O resultado obtido com a análise do teste-reteste evidenciou que ocorreu consistência nas repetições das respostas da PSAS adaptada para o Brasil. Neste estudo, esta análise obteve coeficiente de correlação de 0.928, valor superior à versão original que obteve resultado de 0.8810^(10)^ e à versão francesa, 0.8439^(22)^, porém similar à versão iraniana e turca^(21,33)^. Assim, pode-se, então, afirmar que a PSAS adaptada para o Brasil, além de consistente e confiável, também apresenta estabilidade ao longo do tempo^(20,42)^.

Os valores da análise da validade convergente evidenciaram correlações positivas e estatisticamente significantes entre a versão adaptada da PSAS para o Brasil e os questionários utilizados. Dentre as análises, identificou-se que a correlação mais forte ocorreu com o formulário IDATE-T (traço) (0,515 - correlação significativa no nível 0,01). Tal foumulário avalia a disposição geral à ansiedade, enquanto o IDATE-E (estado) verifica a ansiedade situacional^(22)^. Resultado semelhante também foi encontrado no estudo de Fallon et al.^(10)^ e Duran^(21)^ e, além de indicar boa validade convergente, pode estar associado ao fato de que a ansiedade no pós-parto poderia estar relacionada a uma exacerbação de vulnerabilidades psicológicas, como a vulnerabilidade à própria ansiedade, em face dos múltiplos ajustes psicológicos que a maternidade traz consigo^(43)^. Já a correlação positiva com a EPDS confirma que ansiedade e depressão podem coexistir no pós-parto^(10,44)^.

### Limitações do estudo

Considerando a amostra deste estudo, reconhece-se que uma limitação pode estar relacionada à razão entre o número de observações e variáveis, que foi 5:1. Embora esta razão atenda à recomendação mínima de 5 observações mínimas por variável^(18)^, está no limite inferior, pois a literatura sugere uma amostra com 500 ou mais casos, sempre que possível, para garantia de decisões confiáveis com um mínimo de 200 respondentes^(45,46)^. Tal aspecto pode gerar outra limitação relacionada à generalização dos resultados uma vez que a capacidade de generalizar os resultados para uma população maior pode ser limitada se a amostra não for grande o suficiente para capturar a variabilidade na população. As autoras reconhecem como outra possível limitação o fato de o reteste ter sido realizado por contato telefônico, pois entrevistas por telefone estão sujeitas a interrupções e ruídos de fundo que podem afetar a qualidade da comunicação bem como problemas técnicos como má qualidade de sinal, quedas de chamada ou equipamentos inadequados que podem comprometer a qualidade da entrevista telefônica.

### Contribuições para as áreas de enfermagem, saúde ou políticas públicas

Investigar a ansiedade no período pós-parto é uma intervenção necessária. Nesse sentido, o fornecimento de uma ferramenta específica para este fim poderá contribuir com melhorias no processo de identificação deste agravo e trazer avanços nas pesquisas, contribuindo, assim, para o melhor planejamento do cuidado da saúde mental no período pós-parto.

## CONCLUSÕES

A versão brasileira da *Postpartum Specific Anxiety Scale* (PSAS-BR) indicou evidências de validade da estrutura interna. Assim sendo, pode-se considerar a PSAS-BR como uma ferramenta adequada e útil para uso na prática clínica de investigação da ansiedade nos primeiros seis meses após o parto. Portanto, considera-se que este instrumento poderá ser relevante para ser utilizado no âmbito da saúde mental no puerpério, pois poderá beneficiar a prática dos profissionais de saúde quanto à promoção da saúde, em especial enfermeiros que lidam diária e continuamente com esse público.
